# Supplementary material for: A qualitative interview study on quality of life and ageing experiences of autistic adults
Source: Commun Psychol. 2024 Oct 25;2:99. doi: 10.1038/s44271-024-00142-0 (PMC11512050; doi:10.1038/s44271-024-00142-0)
Supplement: Supplementary file 2 — Reporting Summary [file 44271_2024_142_MOESM2_ESM.pdf]

nature portfolio

Corresponding author(s): Hannah E. Viner

Last updated by author(s): Sep 30, 2024

Reporting Summary

Nature Portfolio wishes to improve the reproducibility of the work that we publish. This form provides structure for consistency and transparency in reporting. For further information on Nature Portfolio policies, see our Editorial Policies and the Editorial Policy Checklist.

Please do not complete any field with "not applicable" or n/a. Refer to the help text for what text to use if an item is not relevant to your study.
For final submission: please carefully check your responses for accuracy; you will not be able to make changes later.

Statistics

For all statistical analyses, confirm that the following items are present in the figure legend, table legend, main text, or Methods section.

n/a	Confirmed	
☒	☐	The exact sample size (*n*) for each experimental group/condition, given as a discrete number and unit of measurement
☒	☐	A statement on whether measurements were taken from distinct samples or whether the same sample was measured repeatedly
☒	☐	The statistical test(s) used AND whether they are one- or two-sided<br>*Only common tests should be described solely by name; describe more complex techniques in the Methods section.*
☒	☐	A description of all covariates tested
☒	☐	A description of any assumptions or corrections, such as tests of normality and adjustment for multiple comparisons
☒	☐	A full description of the statistical parameters including central tendency (e.g. means) or other basic estimates (e.g. regression coefficient) AND variation (e.g. standard deviation) or associated estimates of uncertainty (e.g. confidence intervals)
☒	☐	For null hypothesis testing, the test statistic (e.g. *F*, *t*, *r*) with confidence intervals, effect sizes, degrees of freedom and *P* value noted<br>*Give P values as exact values whenever suitable.*
☒	☐	For Bayesian analysis, information on the choice of priors and Markov chain Monte Carlo settings
☒	☐	For hierarchical and complex designs, identification of the appropriate level for tests and full reporting of outcomes
☒	☐	Estimates of effect sizes (e.g. Cohen's *d*, Pearson's *r*), indicating how they were calculated

Our web collection on statistics for biologists contains articles on many of the points above.

Software and code

Policy information about availability of computer code

Data collection	N/A
Data analysis	NVivo version 14 was used to analyse the interview data.

For manuscripts utilizing custom algorithms or software that are central to the research but not yet described in published literature, software must be made available to editors and reviewers. We strongly encourage code deposition in a community repository (e.g. GitHub). See the Nature Portfolio guidelines for submitting code & software for further information.

Data

Policy information about availability of data

All manuscripts must include a data availability statement. This statement should provide the following information, where applicable:

- Accession codes, unique identifiers, or web links for publicly available datasets
- A description of any restrictions on data availability
- For clinical datasets or third party data, please ensure that the statement adheres to our policy

The data used in this study are not publicly available as they contain information that could compromise the privacy of research participants.

Human research participants

Policy information about studies involving human research participants and Sex and Gender in Research.

Reporting on sex and gender	We report participant gender (based on self-report).
Population characteristics	All participants are aged 40 or over and have a (self-reported) clinical diagnosis of autism.
Recruitment	Flyers were shared in the UK (via social media, a diagnostic service, and personal contacts) and Luxembourg (via an autism organisation). Inclusion criteria were having a clinical diagnosis of autism (self-reported) and being a resident of the UK or Luxembourg. We address the potential impact of differing recruitment strategies in the limitations section of the paper.
Ethics oversight	Ethical approval for this project was granted by the University of Luxembourg Ethics Committee (ERP 23-035 QoLAA).

Note that full information on the approval of the study protocol must also be provided in the manuscript.

Field-specific reporting

Please select the one below that is the best fit for your research. If you are not sure, read the appropriate sections before making your selection.

☐ Life sciences ☒ Behavioural & social sciences ☐ Ecological, evolutionary & environmental sciences

For a reference copy of the document with all sections, see nature.com/documents/nr-reporting-summary-flat.pdf

Behavioural & social sciences study design

All studies must disclose on these points even when the disclosure is negative.

Study description	This is a qualitative interview study.
Research sample	All participants are aged 40 or over and have a (self-reported) clinical diagnosis of autism. The final sample consists of 16 adults (n = 9 UK, n = 7 Luxembourg)
Sampling strategy	We used a convenience sample. We used the "information power" model (Malterud, Siersma & Guassora.,2015) to guide sample size. Furthermore our extensive recruitment efforts did not yield further participants. Data saturation was not considered as it does not align with the values and assumptions of reflexive thematic analysis (Braun & Clarke, 2021).
Data collection	We used a semi-structured interview guide focused on participants' personal views and experiences of ageing and QoL. Interviews were conducted via video conferencing (n = 2 Luxembourg, n = 9 UK), phone (n = 2 Luxembourg) and in-person (n = 3 Luxembourg).
Timing	Interviews were conducted between August 2023 and January 2024
Data exclusions	No data were excluded.
Non-participation	N = 3 participants (n = 2 UK, n = 1 Luxembourg) provided consent but dropped out as they did not meet inclusion criteria (n = 1), were too busy (n = 1), or did not respond to follow-up emails (n = 1).
Randomization	N/A

Reporting for specific materials, systems and methods

We require information from authors about some types of materials, experimental systems and methods used in many studies. Here, indicate whether each material, system or method listed is relevant to your study. If you are not sure if a list item applies to your research, read the appropriate section before selecting a response.

Materials & experimental systems

n/a	Involved in the study
☒	☐ Antibodies
☒	☐ Eukaryotic cell lines
☒	☐ Palaeontology and archaeology
☒	☐ Animals and other organisms
☒	☐ Clinical data
☒	☐ Dual use research of concern

Methods

n/a	Involved in the study
☒	☐ ChIP-seq
☒	☐ Flow cytometry
☒	☐ MRI-based neuroimaging

This checklist template is licensed under a Creative Commons Attribution 4.0 International License, which permits use, sharing, adaptation, distribution and reproduction in any medium or format, as long as you give appropriate credit to the original author(s) and the source, provide a link to the Creative Commons license, and indicate if changes were made. The images or other third party material in this article are included in the article's Creative Commons license, unless indicated otherwise in a credit line to the material. If material is not included in the article's Creative Commons license and your intended use is not permitted by statutory regulation or exceeds the permitted use, you will need to obtain permission directly from the copyright holder. To view a copy of this license, visit http://creativecommons.org/licenses/by/4.0/
